# Intracellular bottlenecking permits no more than three tomato yellow leaf curl virus genomes to initiate replication in a single cell

**DOI:** 10.1371/journal.ppat.1011365

**Published:** 2023-05-01

**Authors:** Ruifan Ren, Limin Zheng, Junping Han, Camila Perdoncini Carvalho, Shuhei Miyashita, Deyong Zhang, Feng Qu

**Affiliations:** 1 Longping Branch, College of Biology, Hunan University, Changsha, China; 2 Department of Plant Pathology, The Ohio State University, Wooster, Ohio, United States of America; 3 Hunan Plant Protection Institute, Changsha, China; 4 Graduate School of Agricultural Science, Tohoku University, Sendai, Japan; Agriculture and Agri-Food Canada, CANADA

## Abstract

Viruses are constantly subject to natural selection to enrich beneficial mutations and weed out deleterious ones. However, it remains unresolved as to how the phenotypic gains or losses brought about by these mutations cause the viral genomes carrying the very mutations to become more or less numerous. Previous investigations by us and others suggest that viruses with plus strand (+) RNA genomes may compel such selection by bottlenecking the replicating genome copies in each cell to low single digits. Nevertheless, it is unclear if similarly stringent reproductive bottlenecks also occur in cells invaded by DNA viruses. Here we investigated whether tomato yellow leaf curl virus (TYLCV), a small virus with a single-stranded DNA genome, underwent population bottlenecking in cells of its host plants. We engineered a TYLCV genome to produce two replicons that express green fluorescent protein and mCherry, respectively, in a replication-dependent manner. We found that among the cells entered by both replicons, less than 65% replicated both, whereas at least 35% replicated either of them alone. Further probability computation concluded that replication in an average cell was unlikely to have been initiated with more than three replicon genome copies. Furthermore, sequential inoculations unveiled strong mutual exclusions of these two replicons at the intracellular level. In conclusion, the intracellular population of the small DNA virus TYLCV is actively bottlenecked, and such bottlenecking may be a virus-encoded, evolutionarily conserved trait that assures timely selection of new mutations emerging through error-prone replication.

## Author summary

An important unresolved issue in virus life cycles is how natural selection acts on individual virus copies occupying the same cell. Unlike cellular organisms in which a chromosome harboring an advantageous or deleterious mutation usually shares the hosting cells with no more than one homologous sister chromosome, viruses could potentially reproduce with numerous genome copies per cell, permitting sharing of protein products, thereby greatly diminishing phenotypic impacts of otherwise eventful mutations. Previous investigations suggest that (+) RNA viruses solve this problem by bottlenecking the number of replicating genome copies in each cell to low single digits. The current study reveals strikingly similar intracellular population bottlenecks for a small DNA virus. Further mechanistic interrogations could avail the virus-encoded bottleneck-enforcing apparatus as targets for antiviral therapy and prevention.

## Introduction

The replication processes of many viruses are highly error-prone, causing most descendant viral genome copies to differ from their parents by a minimum of one mutation [1–5]. While these mutations are mostly phenotypically neutral or near neutral, lethal mutations do occur and can reach high numbers, given that millions of new viral genome copies are frequently produced in each cell. Loss-of-function mutations in viral proteins essential for the replication of viral genomes, if not promptly isolated and purged, pose serious threat to the survival of the cognate virus population. This is because the mutation-containing “cheater” genomes could steal the corresponding mutation-free proteins produced by sister genome copies in the same cell to support their own replication. More ominously, absent of diligent surveillance, similar cheater mutants arise ceaselessly as the replication reiterates. Together these mutant genomes could quickly reproduce themselves to dominance, and steadily dilute out the genome copies still producing mutation-free proteins, ultimately obliterating the cognate viral population [1,2,6,7].

The same challenge also applies to positive selection responsible for enriching beneficial viral mutations. By definition, positive selection feeds the phenotypic gain arisen from a beneficial mutation back to the very genome copy carrying this mutation, causing it to have more surviving descendants than its sister copies. However, such exclusive phenotype-to-genotype feedback is difficult to achieve if mutation-endowed phenotypic gains, most frequently in the form a fitter protein, must be shared among numerous genome copies in the same cell, with the mutation-carrying copy being just one of them. This challenge is further exacerbated by the fact that most beneficial mutations confer minimal phenotypic gains, which could be easily lost if not promptly selected. Therefore, the Darwinian law of natural selection predicts that successful viruses must constrain the number of the reproductive genome copies per cell in order to minimize phenotype cheating or sharing. However, exactly how this is accomplished remains poorly understood.

Recent observations by us and others prompted a new model that explains how plus-strand (+) RNA viruses achieve reproductive isolation [8–11]. Briefly, this Bottleneck, Isolate, Amplify, Select (BIAS) model postulates that multiple genome copies of the same virus, upon penetrating the same cell, cooperate to erect intracellular population bottlenecks from which very few copies (as few as one) of the viral genome could escape to initiate replication. Due to the stochastic nature of the bottlenecks, a viral genome copy with a lethal mutation has the same chance as other sister copies to escape the bottlenecks and use replication proteins produced by the latter to initiate replication. However, should this occur, its replication would lead to the amplification of descendants that all contain the same lethal mutation. Upon invading a fresh cell collectively, such a mutant genome lineage would then be forced to bear the consequence of the lethal mutation, because the mutation-complementing sister genomes would now be absent (or inadequate if the bottleneck size is >1), leading to the elimination or drastic suppression of viral genome copies bearing lethal mutations.

**Fig 1:**
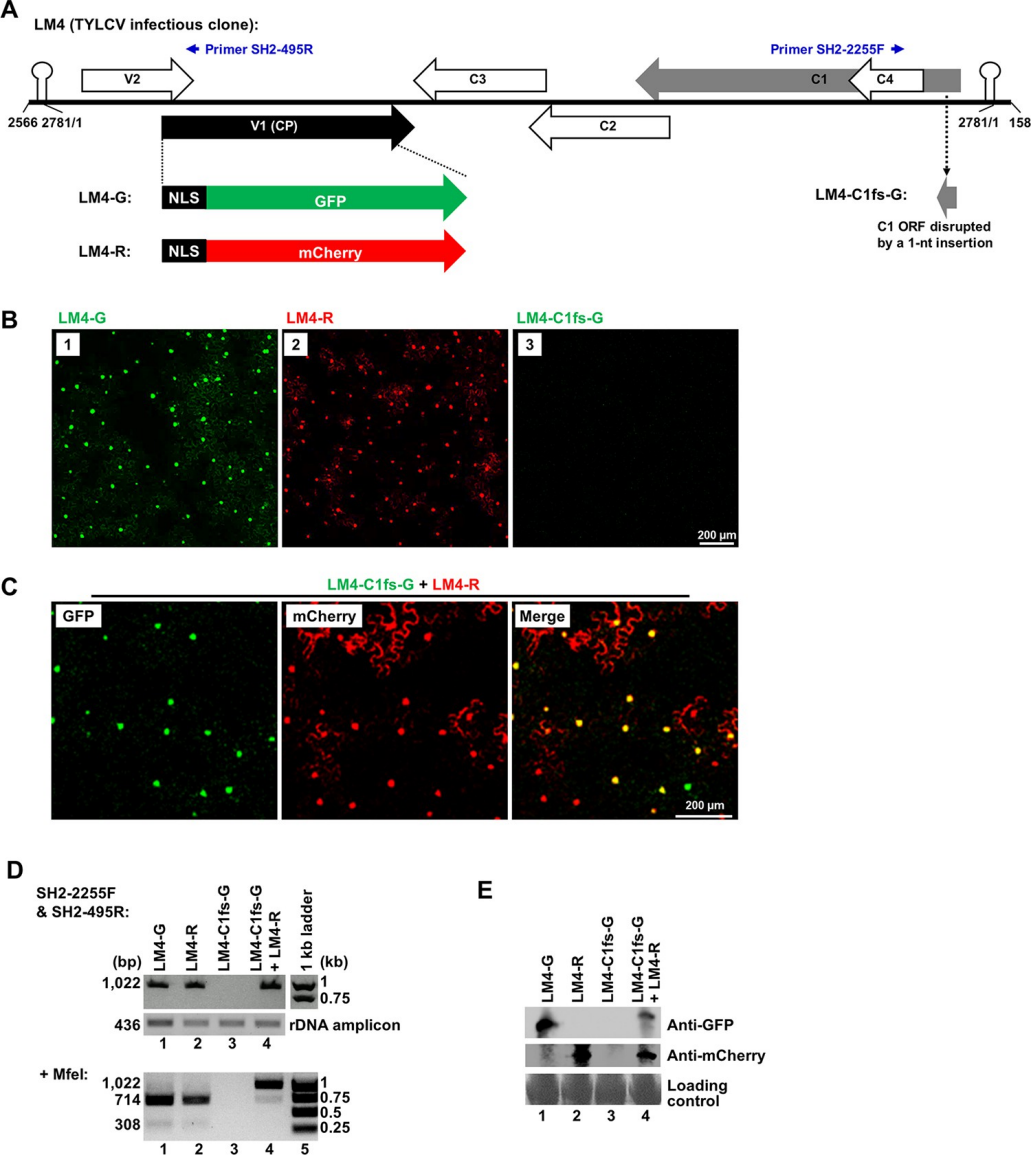
TYLCV-derived replicons LM4-G and LM4-R replicate to produce fluorescent signals that are predominantly localized to nuclei of *N*. *benthamiana* cells. **A**. Schematic depiction of the 2,781-nt TYLCV genome (isolate SH2) in linearized, double-stranded form inserted in a binary shuttle plasmid (pAI. See Materials and Methods for details). Note that nt positions 2566–2781 (256 nt) and 1–158 (158 nt) were duplicated at 5’ and 3’ ends, respectively. The large arrows depict various TYLCV-encoded proteins. The V1 ORF, which was modified to accommodate GFP and mCherry insertions in LM4-G and LM4-R, is highlighted as solid black. The 65-aa N-terminal nuclear localization signal (NLS) of V1 was retained as N-terminal fusions of GFP and mCherry. The C1 ORF was also modified in one of the constructs (LM4-C1fs-G) and is depicted as a gray arrow. Also note the small blue arrows on the top, denoting two primers (SH2-495R and SH2-2255F) used for specific detection of circularized form of the replication-generated viral DNA. **B**. Replication-dependent expression of GFP and mCherry from LM4-G and LM4-R, respectively. The LM4-C1fs-G mutant failed to express GFP as a result of C1 disruption. Bar = 200 μm. **C**. Complementation of LM4-C1fs-G by co-delivered LM4-R. **D**. Verification of replication of LM4-G and LM4-R, as well as replicational complementation of LM4-C1fs-G by LM4-R, with PCR (top) coupled with MfeI digestion. A 436-bp ribosomal DNA (rDNA) fragment was simultaneously amplified (18 PCR cycles) to serve as the control to ensure the samples contained similar amounts of template DNA. Sequences of the rDNA primers are available upon request. The MfeI site present in wildtype (wt) C1 coding sequence was abolished in LM4-C1fs-G by the one-nt insertion within the site that also disrupted the C1 ORF. **E**. Western blotting detection of GFP and mCherry proteins expressed by the LM4-G and LM4-R replicons.

Obviously, the same BIAS arrangement also assures positive selection that proliferates beneficial mutations as soon as they emerge. Nevertheless, while the BIAS model is amply supported by evidence derived from (+) RNA viruses, it is not known whether it also applies to DNA viruses. To address this knowledge gap, here we examined a small DNA virus to determine whether its populations also encountered narrow reproductive bottlenecks in infected cells. We adopted tomato yellow leaf curl virus (TYLCV) as the model virus for this study. TYLCV is a member of the genus *Begomovirus*, family *Geminiviridae*, with a single-stranded, circular DNA genome of approximately 2,800 nucleotides (nt), encoding at least six proteins [12–14]. The four genes on the complementary strand of the genome are early expressing, encoding proteins (C1-C4) that participate in various aspects of viral genome replication, transcriptional activation, and host defense neutralization (Fig 1A) [13,15]. In particular, C1 is absolutely required for the rolling circle replication of TYLCV genome [16–18]. On the other hand, the two genes on the viral strand are both late expressing, dependent on successful genome replication. They encode V1 and V2, which are capsid protein (CP) and suppressor of RNA silencing, respectively (Fig 1A). V1 (CP) is not essential for intracellular replication of TYLCV [19], but needed for the intra- and inter-cellular movement of the virus [20]. In the current report, the genome of the TYLCV SH2 isolate was modified to encode nuclearly localized fluorescent proteins in place of V1, permitting convenient tracking of viral replication in single cells. We found that TYLCV intracellular populations were constrained by stringent bottlenecks that limit replicating genome copies to no more than three in each cell. Furthermore, TYLCV genomes that entered cells early exerted strong superinfection exclusion (SIE) against those entering merely 24 hours later. Together these data suggest that small DNA viruses like TYLCV may also utilize a strategy similar to BIAS to enable timely natural selection of new mutations.

## Results

### TYLCV replicons readily replicate in cells of *Nicotiana benthamiana* leaves

The SH2 isolate of TYLCV has a single-stranded, circular DNA genome of 2,781 nt (Fig 1A). Previous studies by others established that expression of TYLCV V1 gene, encoding viral CP, occurs in a replication-dependent manner [21–23]. We hence reasoned that its replication could be monitored microscopically by replacing part of the CP coding sequence with that of green fluorescence protein (GFP) or the red fluorescent mCherry. Such replacements gave rise to two TYLCV replicons designated as LM4-G and LM4-R, respectively (Fig 1A). Note that the V1 and V2 genes partially overlap. As a result, the V2-overlapping portion of V1 was retained in LM4-G and LM4-R in the form of N-terminal fusions to GFP and mCherry. This 65 amino acid (aa) N-terminal fusion harbors a nuclear localization signal (NLS) that routes most of the GFP and mCherry signals to the cell nuclei, permitting convenient quantification of TYLCV-replicating cells. Also worth noting is that CP replacement by GFP/mCherry abolished viral cell-to-cell movement, thus restricting the replication of LM4-G and LM4-R in single cells.

To test their replicability, the LM4-G and LM4-R replicons were cloned into the pAI101 binary vector [24]. The resulting constructs, still referred to as LM4-G and LM4-R for simplicity, were transformed into *Agrobacterium tumefaciens* (strain C58C1; simplified as agro hereafter), suspensions of which were pressure-infiltrated into leaves of *N*. *benthamiana* plants (agro-infiltration). Both replicons produced abundant, strongly fluorescent signals that were predominantly located in the nuclei of epidermal cells, though modest levels of fluorescence were also detected in the cytoplasm of some cells (Fig 1B, panels 1 and 2). This result demonstrated that both LM4-G and LM4-R replicated robustly in epidermal cells of *N*. *benthamiana*.

To further confirm that expression of fluorescent proteins from LM4-G and LM4-R was dependent on viral replication, we produced the LM4-C1fs-G mutant by inserting one nt early in the C1 open reading frame (ORF) of LM4-G, causing the C1 frame to shift and stop prematurely (Fig 1A, bottom right). This mutant no longer produced any GFP fluorescence (Fig 1B, panel 3). Crucially, the C1 defect of LM4-C1fs-G was successfully complemented by wildtype C1 proteins provided *in trans* from the co-delivered LM4-R replicon, causing most of treated cells to express both GFP and mCherry (Fig 1C).

Replication of LM4-G and LM4-R, as well as replicational complementation of LM4-C1fs-G by LM4-R, were further verified with two additional approaches. First, 15 cycles of polymerase chain reaction (PCR) were carried out with a pair of primers (SH2-2255F and SH2-495R; Fig 1A) that could only produce a fragment from replication-generated circular viral genomes. As illustrated in Fig 1A, these two primers annealed to positions outside of the duplicated termini (nt positions 2,566–2,781 at the left terminus, 1–158 at the right terminus) of LM4, and oriented away from each other in the linear genome inserted in the replicon constructs, hence should not generate a fragment from the non-replicating replicon DNA. As expected, 15 cycles of PCR were sufficient to produce a 1,022 base pair (bp) fragment from DNA samples extracted from tissues receiving LM4-G, LM4-R, and LM4-C1fs-G plus LM4-R (Fig 1D, top panel, lanes 1, 2, 4), but not LM4-C1fs-G alone (lane 3). To ensure similar amounts of template DNA were present in all samples, an 18-cycle PCR was simultaneously performed to amplify a 436-bp ribosomal DNA (rDNA) fragment (Fig 1D, middle panel).

The one-nt insertion in LM4-C1fs-G also disrupted an MfeI site (CAATTG). We thus used MfeI digestion of the PCR products to verify the replicational complementation of LM4-C1fs-G by LM4-R. As shown in Fig 1D, bottom panel, the LM4-G and LM4-R-derived PCR products, upon MfeI digestion, were split into two smaller fragments of 714 bp and 308 bp, respectively (lanes 1 and 2). By contrast, the majority of PCR product derived from the LM4-C1fs-G plus LM4-R sample remained undigested (lane 4), thus verifying successful replication of LM4-C1fs-G in the presence of LM4-R.

We next verified the replication-dependent expression of GFP and mCherry proteins with Western blotting, using antibodies that specifically reacted to either one, but not both. As shown in Fig 1E, GFP protein was detected in samples with LM4-G, LM4-C1fs-G plus LM4-R (top panel, lanes 1 and 4), but not in those with LM4-R or LM4-C1fs-G alone (lanes 2 and 3). Similarly, mCherry protein was detectable in samples containing LM4-R and LM4-C1fs-G plus LM4-R (Fig 1E, middle panel, lanes 2 and 4). Together these experiments established LM4-G and LM4-R as robust TYLCV replicons suitable for examining the intracellular bottlenecking of viral populations.

### Agro-infiltration enables five or more agro cells to deliver transfer DNA (T-DNA) into a single *N*. *benthamiana* cell

To adopt LM4-G and LM4-R for investigating the intracellular population dynamics of TYLCV, we first must determine whether these two replicons, when delivered via agro-infiltration, could co-enter a sufficiently high percentage of *N*. *benthamiana* cells. Note that successful agro-infiltration-mediated delivery of two or more constructs into the same plant cells has been widely reported, most notably for bi-fluorescence complementation experiments (BIFC; e.g. ref 25); but also for studying plant viruses with multi-partite genomes [26,27]. However, these earlier studies did not carefully evaluate the frequency of cells internalizing all co-delivered constructs, and conditions for maximizing same-cell internalization.

**Fig 2:**
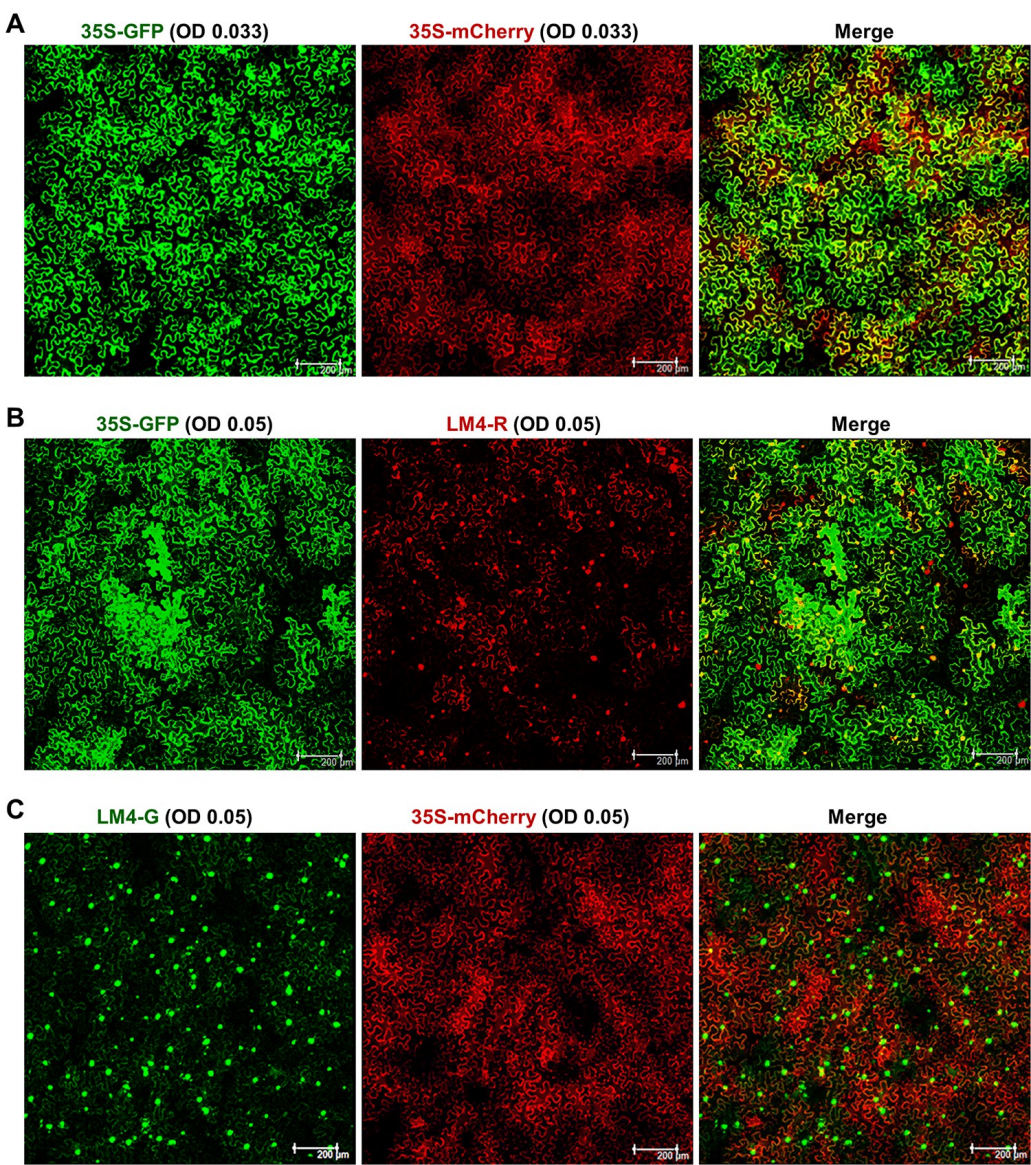
*A*. *tumefaciens* (agro) harboring two different constructs deliver both constructs into same *N*. *benthamiana* cells at very high efficiencies even at the low OD_600_ values of 0.033–0.05. **A**. Both 35-GFP and 35-mCherry were non-replicating constructs that expressed GFP and mCherry transiently upon entering cells. **B** and **C**. Agros harboring LM4-R and LM4-G replicons were mixed with those containing 35S-GFP and 35S-mCherry, respectively. Prior to mixing, all agro suspensions were diluted to OD 0.1; and all mixtures contained an equal volume of all constituents.

We began our assessment using 35S-GFP and 35S-mCherry, two non-replicating constructs that drove transient expression of GFP and mCherry with the strong 35S promoter of cauliflower mosaic virus (CaMV). A third construct expressing the p19 silencing suppressor of tomato bushy stunt virus (TBSV) was also included to counteract the silencing of GFP and mCherry transcripts. Suspensions of agro cells harboring 35S-GFP, 35S-mCherry, and p19 were first diluted to OD_600_ = 0.1, and the diluted agro suspensions were mixed at a volume ratio of 1:1:1. As a result, the final concentration of each agro line was OD 0.033 (Fig 2A). As shown in Fig 2A, both GFP and mCherry were detected in close to 100% of the cells, with more than 95% cells expressing both. This high level of co-expression suggested that each of the *N*. *benthamiana* cells must have received 35S-GFP and/or 35S-mCherry constructs translocated from 5 or more independent agro cells. (We recognize that only the T-DNA portion of a binary construct was mobilized into plant cells by agros; thus, the somewhat inaccurate use of the term “construct” here is purely for simplicity)

To explain the rationale for the above conclusion, we first imagine just one agro cell succeeded in engaging every *N*. *benthamiana* cell. Given the inoculum contained an equal amount of 35S-GFP and 35S-mCherry-carrying agro cells, 50% of plant cells would have received 35S-GFP, the remaining 50% 35S-mCherry. Put it differently, any given single plant cell should have a 50% chance to receive either 35S-GFP or 35S-mCherry, but never both. Using the symbols “g” and “r” to represent 35S-GFP and 35S-mCherry, respectively, these chances could be expressed with the following equation (Table 1, row 1):

50%g+50%r=0.5g+0.5r

We next consider the scenario of two agro cells engaging each plant cell. Given that cells of *N*. *benthamiana* vastly outsizes that of agro cells (roughly 150 μm versus 3 μm in lengths, thus 50: 1 length-wise, >50,000: 1 volume-wise), engagement of a plant cell by two agro cells most likely constituted two independent events. Thus, the chances of a cell of receiving two copies of 35S-GFP (g^2^), two copies of 35S-mCherry (r^2^), or one of each (gr) could be expressed as (Table 1, row 2):

$$(0.5g+0.5r)\;X\;50\%g+(0.5g+0.5r)\;X\;50\%r=(0.5g+0.5r)\;X\;(0.5g+0.5r)=0.25g^{2}+0.5gr+0.25r^{2}$$

**Table 1:** Estimating the number of agro cells translocating binary constructs to each plant cell.

Agro cell #	(g = 35S-GFP; r = 35S-mCherry)	Plant cells fluorescing:		
		green	both	red
1	50%g + 50%r = 0.5g + 0.5r	50%	0	50%
2	(0.5g + 0.5r) X (0.5g + 0.5r) = (0.5g + 0.5r)^2^ = 0.25g^2^ + 0.5gr + 0.25r^2^	25%	50%	25%
3	(0.5g + 0.5r)^3^ = 0.125g^3^ + 0.375(g^2^r + gr^2^) + 0.125r^3^	12.5%	75%	12.5%
4	(0.5g + 0.5r)^4^ **= 0.0625g**^**4**^ **+ 0.25(g**^**3**^**r + gr**^**3**^**) + 0.375g**^**2**^**r**^**2**^ **+ 0.0625r**^**4**^	6.25%	87.5%	6.25%
5	(0.5g + 0.5r)^5^ = 0.03125g^5^ + 0.15625(g^4^r + gr^4^) + 0.3125 (g^3^r^2^ + g^2^r^3^) + 0.03125r^5^	3.125%	93.75%	3.125%

Therefore, successful engagement of a single plant cell by two separate agro cells would permit just 50% of plant cells to express both GFP and mCherry, whereas those expressing GFP or mCherry alone would each amount to 25%. Repeating this thought experiment for situations of 3, 4, and 5 agro cells engaging each plant cell, we arrived at three additional equations, listed as rows 3–5 of Table 1. According to these equations, 5 different agro cells penetrating each plant cell would be expected to permit 93.75% of plant cells to express both GFP and mCherry.

Comparing this predicted value with the observed value of >95%, we concluded that at the concentration of OD 0.033 per agro line (OD 0.066 combined), at least 5 agro cells succeeded in translocating their binary constructs into an average *N*. *benthamiana* cell. Note that the actual number was likely even higher because these calculations left out agro cells carrying the p19 construct. Additionally, Oltmanns and colleagues [28] found that 4–20 copies of the same binary construct routinely resided in each agro cell. Even assuming just 3 of these 4–20 copies were mobilized into plant cells by every agro cell, a minimum of 5 agro cells engaging each plant cell would cause an average plant cell to receive 3 X 5 = 15 copies of binary constructs.

The LM4-G and LM4-R-carrying agro cells were expected to behave similarly to those harboring 35S-GFP and 35S-mCherry, because these binary constructs had the same backbone plasmid (pAI101), thus the same essential elements required for T-DNA translocation (e.g. the right and left border motifs); and were transformed into the same agro strain (C58C1). Nevertheless, we next tested the mixed agro-infiltrations for the 35S-GFP & LM4-R pair (Fig 2B), as well as the LM4-G & 35S-mCherry pair (Fig 2C). Note that even though the mCherry encoded by LM4-R was fused with the V1 NLS, cytoplasmic mCherry fluorescence was detected (e.g. Fig 2B, middle), probably due to its high expression levels in the context of viral replication. Also note that the p19-expressing construct was no longer necessary because replicating LM4-G and LM4-R provided the TYLCV-encoded V2 silencing suppressor. Fig 2B and 2C illustrated that these two pairs of agros likewise resulted in co-expression of GFP and mCherry in more than 95% of *N*. *benthamiana* cells, suggesting that at the concentration of OD 0.05 each (OD 0.1 combined), the replicon-carrying agro cells were competent at delivering constructs into plant cells at a minimum ratio of 5 agro cells per plant cell.

We wish to warn against confusing the above conclusions with the bottleneck size estimations to be described below. Although similar probabilistic laws applied to both sets of experiments, the experiments so far served to ensure two or more co-delivered constructs entered the same agro-infiltrated cells at sufficiently high proportions. In these experiments the LM4-G and LM4-R replicons were paired with the non-replicating 35S-mCherry and 35S-GFP, respectively, but not with each other. As a result, potential intracellular bottlenecks exerted by the replicons would exclusively limit the number of replication-initiating LM4-G or LM4-R copies, without affecting expressions launched by 35S-GFP or 35S-mCherry. By contrast, subsequent experiments aimed to resolve whether the successfully internalized LM4-G and LM4-R replicon copies were then bottlenecked inside the cells, blocking most of them from active replication. It is obvious that this latter question could not be convincingly addressed unless efficient same-cell internalization by multiple replicon copies was established first.

### LM4-G and LM4-R co-replicate in less than 65% of cells that receive both constructs

Results of the previous section predicted that a mix of LM4-G and LM4-R-containing agros, each at the concentration of OD 0.05, would likely enable at least 5 individual agro cells to mobilize their binary constructs into each *N*. *benthamiana* cell, furnishing the latter with 15 or more replicon construct copies. Moreover, increasing the agro titers by 10 folds to OD 0.5 would likely further increase the number of replicon genome copies entering each *N*. *benthamiana* cell. Establishment of same-cell co-existence of multiple copies of two replicon constructs equipped us with the ideal system to address whether replicon genome copies in the same plant cell would be subject to intracellular reproductive bottlenecks that prevent all but a few of them from initiating replication.

**Fig 3:**
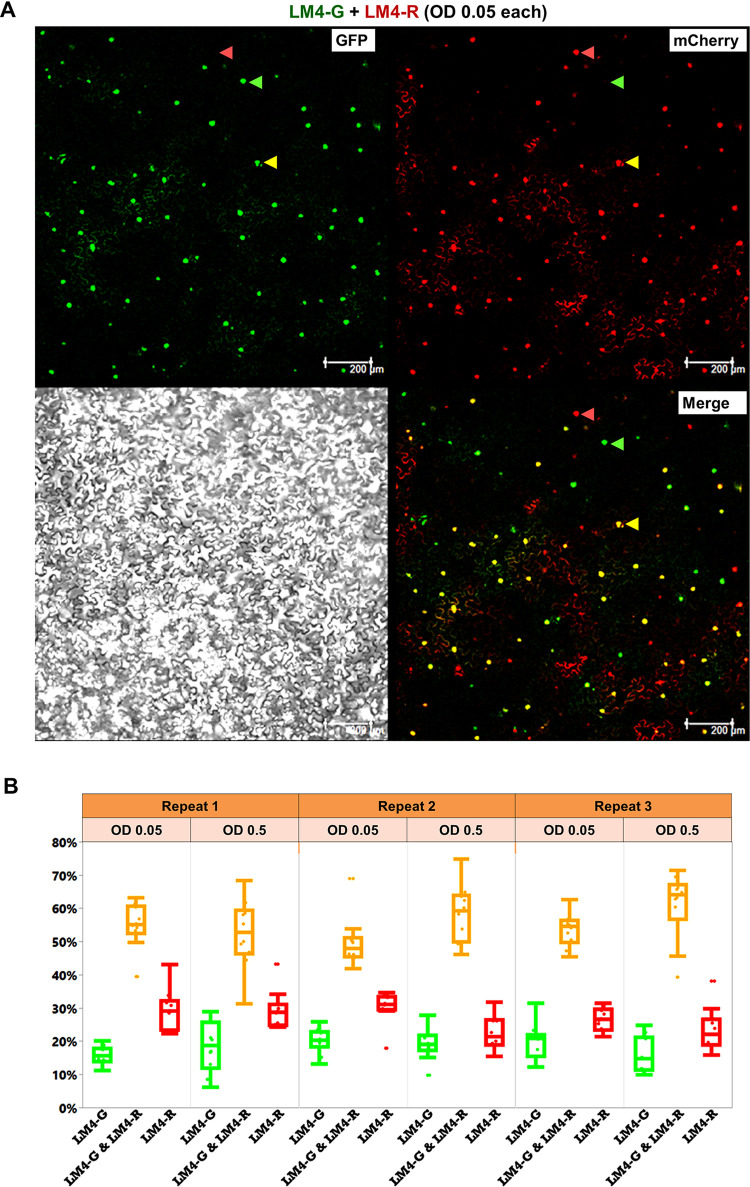
Co-delivery of LM4-G and LM4-R replicons reveals narrow intracellular population bottlenecks of TYLCV. **A**. Images of a typical 2.4 mm^2^ viewing field taken from a leaf section that received both LM4-G and LM4-R, showing nuclei that emit green (GFP) or red (mCherry) fluorescence, or both (Merged), one of each highlighted with arrowheads of different colors. The gray scale image serves as reference for cell sizes, shapes, and boundaries. **B**. Quantification of percentages of cells replicating LM4-G, LM4-R, or both (LM4-G & LM4-R). Shown are box plots derived from numeration data of six experimental groups (three repeats, each with two different agro concentrations, 10 data points per group).

Assuming different replicon copies initiate replication independently of each other, we predicted that if the number of replication-initiating copies was 4 or fewer in each cell, the chance of all of them being either LM4-G or LM4-R alone would be sufficiently high (>6% each) to permit detection, as these cells would express only GFP or mCherry, but not both. We based this prediction on the same probability equations invoked earlier (Table 1), though this time focusing exclusively on replication-initiating (thus bottleneck-escaping) replicon copies. To test this prediction, we prepared two agro mixes that contained an equal amount of LM4-G and LM4-R-containing agro cells, with the concentration of each agro line adjusted to OD 0.05 in the first mix, and OD 0.5 in the second. The infiltrations were carried out on at least six *N*. *benthamiana* leaves, with each leaf divided into two halves: one half receiving the OD 0.05 mix, the other the OD 0.5 mix. Four days after infiltration, 10 2.4-mm^2^ (1.55 X 1.55) viewing fields were randomly chosen for quantification of cells that expressed GFP, mCherry, or both. The entire procedure was repeated three times to ensure reproducibility. Fig 3A shows four confocal images of one viewing field, collected under different light channels. Three of the nuclei were highlighted with green, red, and yellow arrow heads (Fig 3A), representing cells that replicated LM4-G, LM4-R, and both, respectively. Comparing images of different channels (GFP, mCherry, Merge) permitted straightforward differentiation of these cells. Six sets of raw counts of green-, red-, and yellow-fluorescent cells, collected from 10 different viewing fields per set, are provided in the S1 Dataset. For each of the six repeats, the combined cell numbers were then calculated for each of the three categories (green, red, or both), and compiled in Table 2. Their respective percentages were also calculated (Table 2), and used to generate the box plots shown in Fig 3B. As shown in Table 2 and Fig 3B, across the six repeats (three at OD 0.05 and three at OD 0.5), the percentages for cells replicating solely LM4-G ranged from 15.70% to 20.09%, whereas those replicating only LM4-R ranged from 22.56 to 30.14%. Finally, cells replicating both replicons were between 49.77 and 60.87%. These data unveiled a consistent trend of stringent intracellular population bottlenecking that limited the number of replicon genome copies replicating in each cell to low single digits (more in next section).

**Table 2:** Combined counts of green-, red-, and yellow (g &r) fluorescent cells for each of the six repeats, and their respective percentages, obtained with mixed delivery of two independent constructs (LM4-G and LM4-R).

Repeat	Number and fraction of fluorescent cells								g: r ratio
	Green (g)		Red (r)		g & r		Total		
	#	%	#	%	#	%	#	%	
1^st^, OD 0.05	200	15.73	373	29.45	701	54.82	1274	100	45.7: 54.3
1^st^, OD 0.5	231	18.28	356	29.35	628	52.37	1215	100	46.5: 53.5
2^nd^, OD 0.05	336	20.09	498	30.14	834	49.77	1668	100	46.8: 53.2
2^nd^, OD 0.5	293	19.12	344	22.56	876	58.32	1513	100	49: 51
3^rd^, OD 0.05	175	19.94	231	26.5	468	53.56	874	100	47.9: 52.1
3^rd^, OD 0.5	149	15.7	220	23.43	579	60.87	948	100	47.6: 52.4

### The size of TYLCV intracellular population bottlenecks is no larger than three

We next set out to estimate the size of intracellular bottlenecks encountered by the LM4-G and LM4-R replicons. As noted earlier, the equations in Table 1 assumed the two co-delivered constructs (35S-GFP and 35S-mCherry) being similarly competent. However, the data in Table 2 and Fig 3B indicated that in LM4-G & LM4-R mixed infections, LM4-G replicated in a slightly smaller number of cells than LM4-R. Thus, the earlier equations must be adjusted before being adopted for the current purpose. To this end, we calculated a relative LM4-G/LM4-R ratio (g: r) for each of the six repeats (Table 2, right column). These repeat-specific ratios were then averaged to obtain a mean ratio of 47: 53 for cells replicating LM4-G and LM4-R, respectively. The probability equations adjusted using this ratio are listed in Table 3.

**Table 3:** Equations for estimating bottleneck sizes with g: r = 47: 53.

Bottleneck size	Equation	Predicted outcomes		
		green	both	red
1	(0.47g + 0.53r)^1^ = 0.47g + 0.53r	47%	0	53%
2	(0.47g + 0.53r)^2^ = 0.22g^2^ + 0.5gr + 0.28r^2^	22%	50%	28%
3	(0.47g + 0.53r)^3^ = 0.10g^3^ + 0.35g^2^r + 0.40gr^2^ + 0.15r^3^	10%	75%	15%
4	**(0.47g + 0.53r)**^**4**^ **= 0.049g**^**4**^ **+ 0.22g**^**3**^**r + 0.372g**^**2**^**r**^**2**^ **+ 0.28gr**^**3**^ **+ 0.079r**^**4**^	4.9%	87.2%	7.9%

According to Table 3, if just 3 of the replicon copies succeeded in initiating replication in the same cell, meaning an intracellular population bottleneck size of 3, we would detect 75% cells fluorescing yellow, 10% green, and 15% red. Higher percentages of green or red-only cells would indicate a smaller bottleneck size. By comparison, the observed percentages depicted in Table 2 and Fig 3B were 49.77–60.87% yellow, 15.70–20.09% green, and 22.56–30.14% red. These value ranges, with fewer yellow cells but more green and red cells, indicated that the actual bottleneck size must be smaller than 3. On the other hand, an intracellular population bottleneck size of 2 would predict 50% cells being yellow, 22% green, and 28% red. These predicted values were modestly lower than observed for yellow cells, higher for green cells, but nearly as predicted for red cells. Taken together, our data fit the prediction of a bottleneck size between 2 and 3, meaning no more than 3 replicon copies initiated replication in an average *N*. *benthamiana* cell.

To obtain more precise estimates, we assumed that the bottleneck sizes varied among individual cells according to the Poisson distribution, with an average size of *λ*. We then computed *λ* and its standard deviation using a maximum-likelihood method first developed by Miyashita and colleagues [8]. Meanwhile, since LM4-G alone replicated in slightly fewer cells than LM4-R alone, we simultaneously estimated the ratio of g relative to (g + r), designated *r*_g_ in the equations below, for each repeat to account for the effect of replication bias. Specifically, using the variables *λ* and *r*_g_, the expected proportion of LM4-G only cells (*P*_g_), LM4-R only cells (*P*_r_), and co-infected cells (*P*_g+r_), can be expressed as

$$P_{g}=\frac{1}{1-p_{0}}\sum_{k=1}^{\infty }p_{k}∙{r_{g}}^{k},$$

$$P_{r}=\frac{1}{1-p_{0}}\sum_{k=1}^{\infty }p_{k}∙{\left(1-r_{g}\right)}^{k},\;\mathrm{and}$$

$$P_{g+r}=1-P_{g}-P_{r},$$

respectively, where *p*_*k*_ is the probability to have the bottleneck size of *k* following the Poisson distribution with an average size of *λ*:

$$p_{k}=\frac{\lambda^{k}e^{-\lambda }}{k!}.$$

The likelihood *L* (in other words, fitness to the observed data) can be calculated using the observed numbers of LM4-G only cells (*c*_g_), LM4-R only cells (*c*_r_), and co-infected cells (*c*_g+r_) together with their expected proportions *P*_g_, *P*_r_, and *P*_g+r_ as

$$L=\frac{c_{total}!}{c_{g}!c_{r}!c_{g+r}!}{P_{g}}^{c_{g}}{P_{r}}^{c_{r}}{P_{g+r}}^{c_{g+r}}.$$

Then, the most likely estimates for *λ* and *r*_g_ were obtained by searching for *λ* and *r*_g_ that maximize the likelihood *L* (see Materials and Methods and SI Text for code availability information). We allowed different *r*_g_ values for different experimental repeats, because the relative activities of mixed agro suspensions varied slightly. As a result of maximum-likelihood computations, we obtained *λ* values of 2.38 ± 0.05 and 2.60 ± 0.05 for OD 0.05 and 0.5, respectively (Table 4), with the g/(g + r) ratio *r*_g_ ranging from 0.42 to 0.48. This suggests that the size of intracellular TYLCV population bottlenecks was no more than three, and was largely unaffected by a 10-fold increase of the inoculum dose.

**Table 4:** Maximum-likelihood estimates of *λ* and g/(g + r) with LM4-G and LM4-R as separate constructs.

	*λ*	g/(g + r)		
		Repeat 1	Repeat 2	Repeat 3
Estimates (OD = 0.05)	2.38 ± 0.05	0.42 ± 0.01	0.44 ± 0.01	0.46 ± 0.01
Estimates (OD = 0.5)	2.60 ± 0.05	0.43 ± 0.01	0.48 ± 0.01	0.45 ± 0.01

### TYLCV intracellular population bottlenecks remain stringent when the LM4-G and LM4-R replicons are combined in a single construct

**Fig 4:**
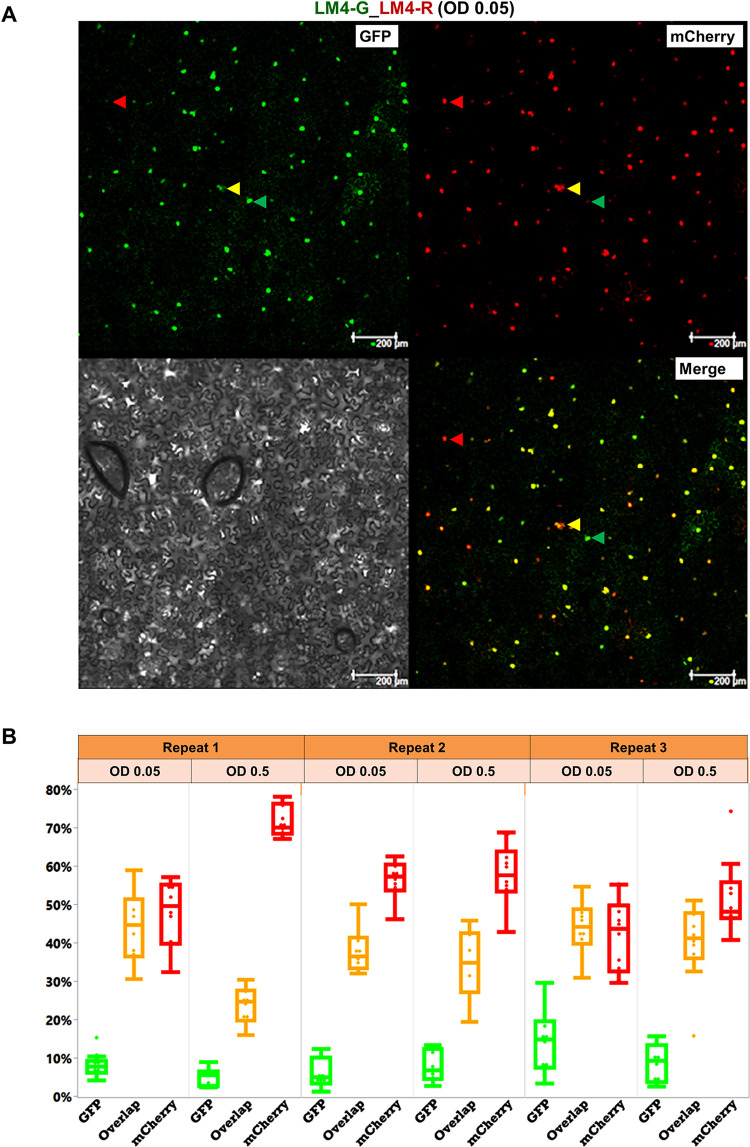
Guaranteeing same-cell internalization of both replicons using a single construct to host both LM4-G and LM4-R. **A**. Images of a typical 2.4 mm^2^ viewing field taken from a leaf section that received the single construct LM4-G_LM4-R, showing nuclei that emit green (GFP) or red (mCherry) fluorescence, or both (Merged), one of each highlighted with arrowheads of different colors. The gray scale image serves as reference for cell sizes, shapes, and boundaries. The intracellular population bottlenecks were again manifest, as indicated by fractions of cell nuclei replicating LM4-G, LM4-R, or both (LM4-G & LM4-R). **B.** Box plots derived from numeration data of six experimental groups (three repeats, each with two different agro concentrations, 10 data points per group).

Earlier experiments indicated that co-delivering LM4-G and LM4-R in the form of mixed agro suspensions, at OD 0.05 each, permitted >95% of *N*. *benthamiana* cells to receive both replicons. Nevertheless, there was still a small chance that a few *N*. *benthamiana* cells received multiple copies of a single replicon construct (meaning LM4-G or LM4-R only), thus distorting the results of bottleneck size estimations. To address this concern, we next assembled a new construct, designated LM4-G_LM4-R, by combining both replicons in the same DNA molecule. The LM4-G_LM4-R construct was then delivered into *N*. *benthamiana* cells at two agro concentrations (OD 0.05 and 0.5), and cell nuclei expressing GFP only, mCherry only, or both were counted for three repeat experiments. As shown in Fig 4, Table 5, and S2 Dataset, this new construct further compromised the relative competitiveness of LM4-G. This notion was supported by maximum-likelihood estimations, which showed that the g/(g + r) ratio *r*_g_ was significantly lower than 0.5 (ranging from 0.13 ± 0.01 to 0.32 ± 0.01; Table 6). Nevertheless, the estimated bottleneck sizes, 2.37 ± 0.07 and 2.20 ± 0.08 for OD 0.05 and 0.5, respectively, were not substantially different from the estimates obtained with two separate constructs (Table 4). Thus, guaranteeing the same cell co-existence of both LM4-G and LM4-R by combining them in the same construct provided further corroboration of earlier estimation of an intracellular bottleneck size of no more than 3.

**Table 5:** Combined counts of green-, red-, and yellow (g &r) fluorescent cells for each of the six repeats, and their respective percentages, obtained with a single construct combining LM4-G and LM4-R.

Repeat	Number and fraction of fluorescent cells							
	Green (g)		Red (r)		g & r		Total	
	#	%	#	%	#	%	#	%
1^st^, OD 0.05	50	8.08	293	47.9	278	44.02	621	100
1^st^, OD 0.5	38	4.97	557	71.65	185	23.38	780	100
2^nd^, OD 0.05	40	5.81	418	56.32	291	37.86	749	100
2^nd^, OD 0.5	68	7.86	490	57.78	293	34.36	851	100
3^rd^, OD 0.05	130	14.2	415	41.87	428	43.93	973	100
3^rd^, OD 0.5	72	8.43	430	51.73	340	39.84	842	100

**Table 6:** Maximum-likelihood estimates of *λ* and g/(g + r) with LM4-G and LM4-R combined in a single construct.

	*λ*	g/(g + r)		
		Repeat 1	Repeat 2	Repeat 3
Estimates (OD = 0.05)	2.37 ± 0.07	0.27 ± 0.01	0.21 ± 0.01	0.32 ± 0.01
Estimates (OD = 0.5)	2.20 ± 0.08	0.13 ± 0.01	0.22 ± 0.01	0.26 ± 0.01

### LM4-G and LM4-R exert strong intracellular SIE to each other

**Fig 5:**
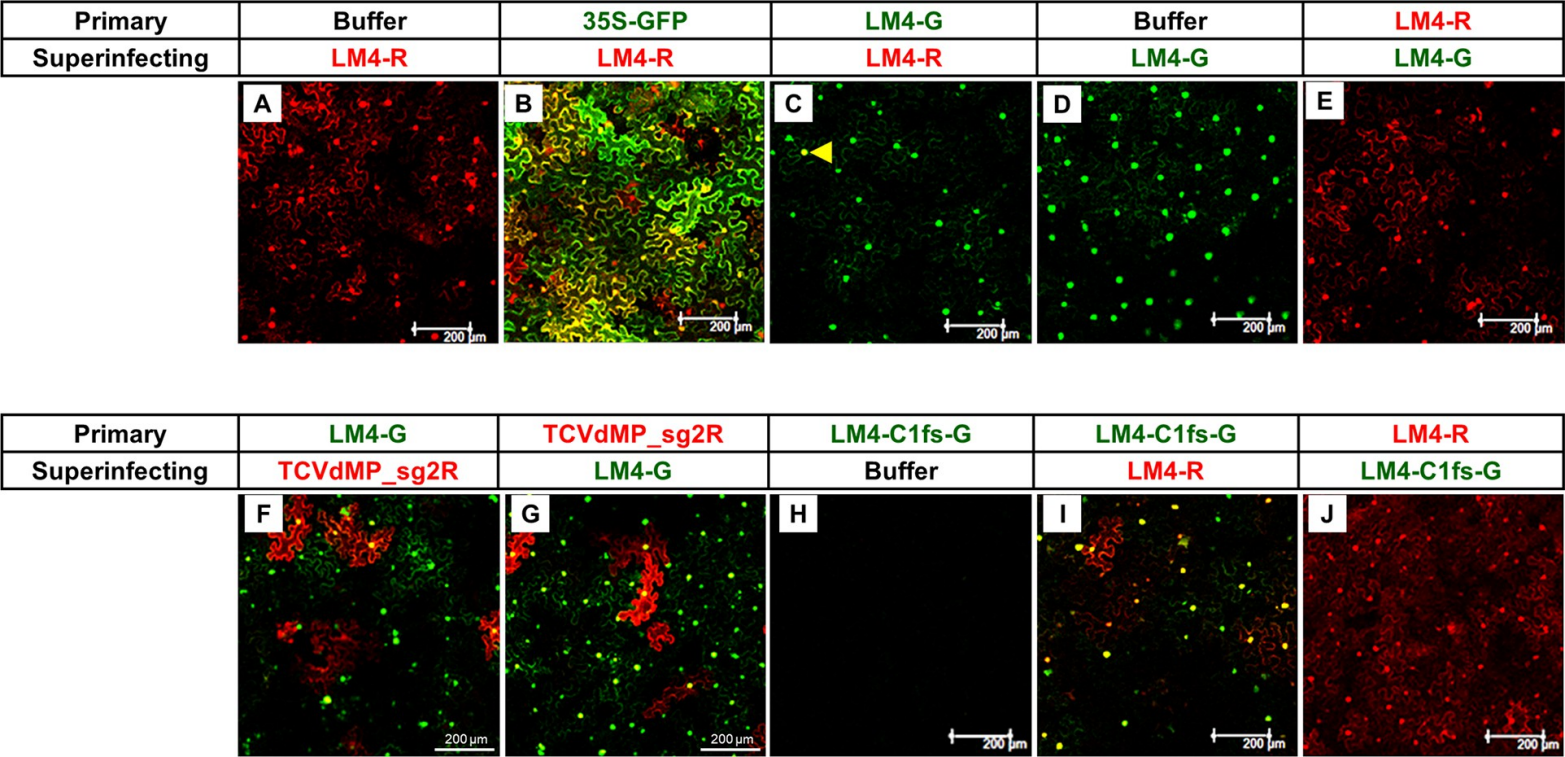
LM4-G and LM4-R exhibit mutual exclusion at the cellular level when delivered sequentially. The primary and superinfecting agros were administered to the same leaf areas with a 24 hour (hr) interval.

We reported earlier that in (+) RNA virus infections SIE manifested intracellular bottlenecking of viral populations [8–11,29,30]. To determine whether the TYLCV replicons also exhibited intracellular SIE, we next used LM4-G and LM4-R to infect *N*. *benthamiana* leaf cells in a sequential manner. As controls, *N*. *benthamiana* leaf cells pre-infiltrated with either infiltration buffer or the 35S-GFP transient expression construct still permitted the replication of superinfecting LM4-R (Fig 5A and 5B). By contrast, a pre-introduced LM4-G exerted a near complete SIE against LM4-R, blocking the latter from replicating in nearly all cells (Fig 5C). Indeed, among more than 1,000 cells inspected in multiple repeat experiments, just one cell was found to replicate the superinfecting LM4-R (Fig 5C, yellow arrowhead). Similarly, a pre-introduced LM4-R completely blocked the replication of LM4-G in the same cells (Fig 5D and 5E).

The mutual SIE between LM4-G and LM4-R could have been caused by exhaustion of cellular resources by the pre-introduced replicon, given the 24-hour time lag between two agro-infiltrations. To address this concern, we tested whether turnip crinkle virus (TCV), a (+) RNA virus, could exert SIE to TYLCV replicons, as TCV replication would be expected to consume large amounts of cellular resources crucial for TYLCV replication as well, such as those needed for transcribing viral mRNAs and translating viral proteins. To this end, we adopted TCVdMP_sg2R, a TCV replicon that expressed mCherry in a replication-dependent manner and, due to a deletion within its movement protein (MP) ORFs, did so exclusively in cells receiving this construct [10]. As shown in Fig 5F and 5G, TCVdMP_sg2R replicated in fewer cells than LM4-G both when pre-introduced and when superinfecting. Nevertheless, in both cases nearly all cells that replicated TCVdMP_sg2R also replicated LM4-G. Therefore, mutual SIE between LM4-G and LM4-R was most likely due to self-exclusion.

We further investigated whether the pre-introduced replicon must replicate itself in order to exert SIE against the superinfecting replicon. To this end, we pre-introduced the defective replicon LM4-C1fs-G, and superinfected the same cells with LM4-R. As shown in Fig 5H and 5I, the defective LM4-C1fs-G was actually complemented by the superinfecting LM4-R, enabling the former to replicate in most cells to produce green-fluorescent nuclei. Intriguingly, some of the cells expressed GFP but not mCherry, suggesting that in these cells, even though the LM4-R replicon construct provided the C1 protein to complement the replication of LM4-C1fs-G, its own replication was blocked by the intracellular reproductive population bottlenecks. Note that in these cells the LM4-R replicon could not be said to be blocked by SIE, because LM4-C1fs-G, despite being pre-introduced, could not replicate until after LM4-R introduction, thus had no chance to exert SIE against the latter.

Consistent with this idea, no complementation occurred when the defective replicon was introduced as a superinfector (Fig 5J). This result, when considered together with the results in Fig 5C and 5E, indicated that by this time (24 hours after introduction of the primary replicon), the intracellular bottlenecks were so firmly established that newly internalized replicons of the same virus no longer had the chance to initiate their own replication. Overall these results suggested that replication of the primary replicon, and/or pre-accumulation of the C1 protein, were needed for robust SIE between TYLCV replicons.

## Discussion

In the current study, we sought to resolve whether multiple copies of the single-stranded, circular genome of a small DNA virus, upon entering the same cell collectively, could all initiate replication or, whether they were subject to intracellular reproductive bottlenecking in a manner similar to (+) RNA viruses [9,10]. To this end, two TYLCV replicons, LM4-G and LM4-R, were created that expressed two different fluorescent proteins (GFP and mCherry) in a replication-dependent manner. Note that these replicons lacked the ability to move cell-to-cell due to the disruption of V1 (CP) ORF. These two replicons were then introduced into leaves of *N*. *benthamiana* plants in the form of mixed agro suspensions to ensure both entered the same cells. Additionally, they were also combined in a single plasmid construct (LM4-G_LM4-R) to guarantee their simultaneous penetration of the same cells. Importantly, GFP and mCherry expressed from LM4-G and LM4-R replicons translocated to the cell nuclei, permitting straightforward numeration of cells that replicated one or both replicons. Counting of tens of thousands of fluorescent cell nuclei, followed by probability computation, led to the conclusion that TYLCV populations were severely bottlenecked intracellularly, permitting no more than three genome copies to commence replication in each cell. Finally, if a molecular mechanism similar to BIAS was responsible for actively enforcing these intracellular bottlenecks, it would likely also explain the cellular level SIE observed between the two TYLCV replicons (Fig 5).

The question of exactly how many genome copies of a DNA virus initiate productive infections in each infected cell have been investigated for several larger DNA viruses with double-stranded DNA genomes [31–33]. In one study, the *Autographa californica* nuclear polyhedrosis virus (AcMNPV) with a 134 kb dsDNA genome was estimated to found cellular infections with approximately 4.3 genome copies per cell [31]. In another, the plant-infecting CaMV with an 8 kb dsDNA genome (though replicating via an RNA intermediate) were estimated to initiate effective cellular infections with 2–13 copies of viral genome, depending on leaf positions and infection stages [32]. It should be noted that the per-cell genome copy numbers established in these earlier studies, designated multiplicity of infection or MOI by the authors, were computed indirectly from results of serial passaging of multiple host individuals [31], or from serial sampling of the systemically infected plant leaves [32]. The authors thus had to make assumptions about the number of cells passed through by the viral genomes. The third study adopted a greatly improved system in which the replication of a recombinant genome of the pseudorabies virus (PRV263) produced three different types of progeny viruses that each expressed a different fluorescent protein [33]. By following the fates of different progeny types in a verifiable number of successive cells, the authors estimated that PRV263 initiated cellular infections with fewer than 7 copies of the genome. However, this study was carried out in cell cultures, which could differ from infected host individuals.

Another more recent study appeared to suggest that faba bean necrotic stunt nanovirus (FBNSV), which divides its genome in 8 different small circular ssDNA segments, may enlist intercellularly trafficked protein products to complete replication cycles of different segments in different cells, hence requiring <1 genome copy per cell on average [27]. However, given that different genome segments of multipartite or segmented viruses could replicate to vastly different levels in different cells [34,35], and the possibility that the in situ hybridization procedure used by the authors may not be sufficiently sensitive to detect low level replication, these findings await further corroborations.

Compared with these earlier studies, our experiments were carried out in cells of intact host plants, where the population dynamics of viral replicons were followed at the single cell level, yielding direct conclusions drawn from primarily infected cells of host plants. Moreover, since the observable output were fluorescent signals emitted by GFP and mCherry, which should have been amplified by replication-dependent transcription and translation, the sensitivity of our approach should far surpass other methods that directly monitored genomic DNA copies, thus generating highly reproducible observations.

Exactly how many genome copies of a virus initiate replication in a single cell is a critical question because it is becoming increasingly clear that many viruses invade cells with not one, but many virions, hence many copies of viral genomes [36]. Consistent with this view, copies of the (+) sense poliovirus genomic RNA were found to direct translation of viral proteins at multiple intracellular sites before commencing replication [37]. On the other hand, even if just one copy of viral genome entered a cell initially and it succeeded in launching replication, we are still faced with the question of how many of the progeny genome copies repeat the replication cycle in the cell of their parent.

The need to address this question is even more acute considering the high error rate of the replication processes of many viruses, estimated to be approximately 10^−4^ for every nt incorporated [7,38,39]. Depending on the size of viral genomes, this error rate translates into approximately one mutation for every new genome copy synthesized. Given the fact that viruses often replicate millions of progeny genome copies in each infected cell, it is inevitable that some of the progeny genome copies contain loss-of-function mutations in essential protein-coding genes. If even 100 of the progeny genomes were permitted to re-replicate in the cell of their own genesis, those with lethal errors would be able to hitchhike on the allelic proteins produced by sister genomes, thus could not be efficiently purged from the virus population.

While it is critically important to weed out lethal mutations in virus populations, similarly important is the need to positively select beneficial mutations. Absent of bottleneck-mediated isolation, a viral genome copy with a beneficial mutation in a protein-coding gene must share the intracellular environment with numerous sister genome copies, thus is unlikely to be the exclusive beneficiary of the mutation-endowed beneficial phenotype. Such a genome copy would then replicate to similar levels as its sister copies, despite the phenotypic advantage it encodes. This scenario is contrary to the real world observations of rapid enrichment of beneficial mutations by viruses, most notably SARS-CoV-2 [40], suggesting intracellular population bottlenecking as a virus-encoded trait to assure both positive and purifying selections [41–44].

Besides serving as a DNA virus model for the BIAS hypothesis, the TYLCV system has additional advantages. To address the challenge of constant emergence of viral mutations, earlier researchers proposed that some of the replication proteins encoded by (+) RNA viruses serve exclusively the very RNA from which they are translated, hence are cis-acting [26,45]. The cis-acting arrangement could explain certain aspects of replicating (+) RNA viruses for which both translation and replication occur in the cytoplasm. However, it would be untenable for DNA viruses like TYLCV. This is because the TYLCV-encoded replication protein (C1) must be translated in the cytoplasm of the infected cells, and then routed back to cell nuclei to orchestrate the rolling circle replication. It is then impossible to differentiate between C1 proteins originated from different TYLCV genome copies.

We hasten to note that this TYLCV-based experimental system is not without limitations. For example, replacing part of the V1 coding sequence with GFP or mCherry interrupted the natural infection process by preventing the intra- and intercellular spread of the viral genomes. Furthermore, as evidenced by the unequal replication of LM4-G and LM4-R, insertion of DNA fragments with different sequences in TYLCV genome likely affected viral replication efficiency to varying extents. Finally, despite our efforts ensuring the same-cell penetration by multiple replicons, it remains to be established exactly how many copies of TYLCV genome are simultaneously translocated into an uninfected cell during natural infections. In short, many gaps remain to be filled with additional investigations.

In summary, with the current study we have demonstrated that populations of the small DNA virus TYLCV are intracellularly bottlenecked in a manner similar to (+) RNA viruses. This finding provides exciting leads for follow-up studies aimed at elucidating the mechanism(s) of the bottlenecking, and examining its potential role in facilitating natural selection in viruses. Outcomes of these follow-up studies will likely avail the bottlenecking machinery as the target for preventive as well as therapeutic interventions of virus diseases of plants, animals, and humans.

## Materials and methods

### Constructs

The original TYLCV infectious clone (isolate SH2. The Genbank accession number is AM282874.1) was kindly provided by Dr. Xueping Zhou of China Institute of Plant Protection [46]. The full-length, double-stranded form of TYLCV genome, plus a 216-bp duplication at the 5’ end, and a 158-bp duplication at the 3’ end, was subcloned into pAI101, a *E*.*coli*-*A*. *tumefaciens* shuttle vector modified from pCambia1300 in our lab [24,47], leading to a new TYLCV infectious clone we call LM4. To create LM4-G and LM4-R, two KpnI sites were introduced into the V1 gene, at positions 307/308 and 881/882 (numbering relative to the full length genome sequence), respectively. The coding sequences of uvGFP and mCherry were then PCR amplified and cloned between the KpnI site using the NEBuilder kit (New England Biolabs). The sequences of LM4, LM4-G and LM4-R encompassing the entire TYLCV DNA (and its modified forms) were verified with Sanger sequencing. Other constructs (p19, 35-GFP, 35S-mCherry) were described previously [30,48,49].

### Agrobacterium infiltration

(agro-infiltration). All DNA constructs destined for testing in *N*. *benthamiana* plants were transformed into electrocompetent A. tumefaciens strain C58C1 via electroporation using the AGR setting on the Bio-Rad Micropulser Electroporator. Briefly, 5 μl of the plasmid DNA was mixed with 40 μl of agro cells and maintained on ice until electroporation. After electroporation, 900 μl of SOB media was added and the suspension was incubated at 28°C for one hour. Selection was carried out on solid Terrific Broth (TB) media containing rifampicin, gentamycin, and kanamycin. Successful introduction of the plasmid was confirmed using colony PCR. A single colony confirmed to have the desired plasmid was used to inoculate 3 ml TB liquid media with the same antibiotics, and incubated overnight at 28°C. The culture was diluted 1:100 with fresh TB liquid media and incubated under the same conditions for another night. The second culture was centrifuged at 4,000 rpm for 20 min, and resuspended in agroinfiltration buffer (10 mM MgCl2, 10 mM MES, and 100 μM acetosyringone). All suspensions were diluted to OD600 = 1 and incubated at 28°C for 3 hours. *Agrobacterium* suspensions were then mixed and introduced into leaves of young *N*. *bethamiana* plants via a small wound, using a needleless syringe.

### Confocal microscopy

Four days after agro-infiltration, leaf discs were collected from the plants. Confocal microscopy was performed at the Molecular and Cellular Imaging Center (MCIC), the Ohio Agricultural Research and Development Center, using a Leica DMI6000 laser confocal scanning microscope. To detect GFP and mCherry fluorescence, sequential excitation at 488 nm and 587 nm was provided by argon and helium-neon 543 lasers, respectively.

### Numeration of the fluorescent nuclei

To count the cells that replicate LM4-G, LM4-R, or both, images of 2.4 mm^2^ (1.55 mm X 1.55 mm) were collected using a 10X lens, from randomly selected leaf sections receiving different combinations of agro suspensions. For samples treated with the mixture of LM4-G and LM4-R, or the combined LM4G_LM4-R construct, images of three separate channels were collected for every selected viewing field to allow for the separate counting of different colored spots (GFP, mCherry, or all spots). For each treatment group, at least 10 images were collected from six different leaves. The fluorescent spots representing nuclei of infected cells were counted using the ImageJ program. The number of nuclei that simultaneously replicated both LM4-G and LM4-R was calculated by subtracting the number of all spots from the sum of green and red spots.

### Statistics and bottleneck size computation

The calculation of sums, means, percentages, standard deviations, were mostly carried out with various tools available through Excel. The box plots were generated with the JMP Pro 16.0.0 software package. Bottleneck size estimations were performed using a maximum likelihood algorithm described in our previous studies [8, 9, 41], based on the R software package ver. 4.1.3 [42]. The script is available as S1 Text in SI Appendix, and downloadable at GitHub (https://github.com/ShuheiMiyashita/Ren_et_al_2022).

## Supporting information

S1 Dataset Raw counts of green (G), red (R), and G&R fluorescent cells for Table 2 and Fig 3B. (XLSX)

S2 Dataset Raw counts of green (G), red (R), and G&R fluorescent cells for Table 5 and Fig 4B. (XLSX)

S1 Text an R script for *λ* and g/(g + r) computation. (DOCX)
