# Supplementary material for: Intracellular bottlenecking permits no more than three tomato yellow leaf curl virus genomes to initiate replication in a single cell
Source: PLoS Pathog. 2023 May 1;19(5):e1011365. doi: 10.1371/journal.ppat.1011365 (PMC10174518; doi:10.1371/journal.ppat.1011365)
Supplement: S1 Text — (DOCX) [file ppat.1011365.s003.docx]

**Supporting Information (SI) for:**

**A small DNA virus initiates replication with no more than three genome copies per cell**

Ruifan Ren^1,2,3,*^, Limin Zheng^2,3,*^, Junping Han^2^, Camila Perdoncini Carvalho^2^, Shuhei Miyashita^4^, Deyong Zhang^1,3,**^, Feng Qu^2,**^

**S1 Text – an R script for *λ* and g/(g + r) computation:**

#An R script for bottleneck size estimation

### Data input

# cgo: no. of cells with GFP only

# cgr: no. of cells with both GFP and mCherry

# cro: no. of cells with mCherry only

# data for separate-vector system (OD 0.05) are used as an example

cgo1 <- 200; cgr1 <- 701; cro1 <- 373 # Rep1

cgo2 <- 336; cgr2 <- 834; cro2 <- 498 # Rep2

cgo3 <- 175; cgr3 <- 468; cro3 <- 231 # Rep3

### Main body of bottleneck size estimation

K <- 30 #maximum number of founders included in the calculation

kv <- NULL

lv <- NULL

for (i in 0:K){

kv <- c(kv,rep(i,i+1))

lv <- c(lv,0:i)

}

klv <- kv-lv

lklv <- lv*klv

ln <- (K+2)*(K+1)/2

## Function for log likelihood for any lambda (bottleneck size) and r (ratio of g to g+r)

FmLL <- function(lr){

lambda <- lr[1]; r1 <- lr[2]; r2 <- lr[3]; r3 <- lr[4];

t <- matrix(rep(0,ln*7),ncol=7)

t[,1] <- kv

t[,2] <- lv

t[,3] <- klv

t[,4] <- lklv

t[,5] <- dpois(t[,1],lambda)*dbinom(t[,2],t[,1],r1)

t[,6] <- dpois(t[,1],lambda)*dbinom(t[,2],t[,1],r2)

t[,7] <- dpois(t[,1],lambda)*dbinom(t[,2],t[,1],r3)

#Rep1

pni1 <- t[1,5]

pgo1 <- sum(t[which(t[,2]>0&t[,3]==0),5])/(1-pni1)

pro1 <- sum(t[which(t[,2]==0&t[,3]>0),5])/(1-pni1)

pgr1 <- 1-pgo1-pro1

#Rep2

pni2 <- t[1,6]

pgo2 <- sum(t[which(t[,2]>0&t[,3]==0),6])/(1-pni2)

pro2 <- sum(t[which(t[,2]==0&t[,3]>0),6])/(1-pni2)

pgr2 <- 1-pgo2-pro2

#Rep3

pni3 <- t[1,7]

pgo3 <- sum(t[which(t[,2]>0&t[,3]==0),7])/(1-pni3)

pro3 <- sum(t[which(t[,2]==0&t[,3]>0),7])/(1-pni3)

pgr3 <- 1-pgo3-pro3

#Log likelihood

LL1 <- dmultinom(c(cgo1,cgr1,cro1),prob=c(pgo1,pgr1,pro1),log=TRUE)

LL2 <- dmultinom(c(cgo2,cgr2,cro2),prob=c(pgo2,pgr2,pro2),log=TRUE)

LL3 <- dmultinom(c(cgo3,cgr3,cro3),prob=c(pgo3,pgr3,pro3),log=TRUE)

LL <- LL1 + LL2 + LL3

-LL

}

## Maximization of log likelihood to find most likely lambda and r

init <- c(5,0.5,0.5,0.5)

FmLL.opt <- optim(init,FmLL, NULL, method="L-BFGS-B", hessian = TRUE, lower=c(0.1,0.01,0.01,0.01), upper=c(10,0.99,0.99,0.99))

FmLL.opt$par # lambda and r estimates

v <- solve(FmLL.opt$hessian)

sd <- sqrt(diag(v))

sd # standard deviations for lambda and r, respectively
